# Working in pairs: male–female village health volunteers supporting maternal health and community engagement in remote and ethnic communities of Lao PDR—a qualitative study

**DOI:** 10.1186/s41182-025-00817-2

**Published:** 2025-10-23

**Authors:** Manami Uehara, Inthanomchanh Vongphoumy, Noudéhouénou Credo Adelphe Ahissou, Tiengkham Pongvongsa, Khampheng Phongluxa, Jun Kobayashi

**Affiliations:** 1 Department of Global Health, Graduate School of Health Sciences, University of the Ryukyus, Okinawa, Japan; 2 Savannakhet Provincial Health Office, Kaysone Phomvihane, Savannakhet, Lao People’s Democratic Republic; 3 Lao Tropical and Public Health Institute, Sisattanack, Vientiane, Lao People’s Democratic Republic

**Keywords:** Community health workers, Male–female pair model, Maternal and child health, Gender norms, Ethnic minority communities

## Abstract

**Background:**

Community health workers (CHWs) are vital for improving maternal and child health (MCH) in underserved settings; however, gender norms often influence their functioning. In the Lao PDR, Village Health Volunteers (VHVs) serve as frontline CHWs in rural areas. Xepon District is a remote border area with Vietnam, characterized by underserved conditions, limited health access, and predominantly ethnic minority populations. The VHV workforce in this district is largely male, which restricts culturally acceptable outreach to women and underscores the need to test a paired male–female model. To address this gender-related limitation, a provincial pilot program introduced male–female VHV/VHW pairs to strengthen MCH outreach.

**Objectives:**

This study aims to explore how the male–female paired VHV model functions in remote ethnic minority communities and its contributions to maternal health promotion.

**Methods:**

A qualitative descriptive study was conducted in 19 pilot villages in Xepon District, Savannakhet Province, from June to October 2024. Semi-structured interviews were held with 42 participants, including mothers, their partners, VHVs, village leaders, health center staff, and district/provincial health officials. Interviews were transcribed, translated, and analyzed using inductive thematic analysis based on Braun and Clarke’s six-phase framework.

**Results:**

Four key themes emerged: (1) building trust and comfort through gender-matched interactions; (2) gender-specific responsibilities and task sharing between paired VHVs; (3) strengthening family support and health system linkages through female VHV/VHWs engagement; and (4) challenges and support needs for strengthening the VHV Program. Female VHV/VHWs played a crucial role in culturally sensitive maternal outreach, whereas male VHVs facilitated engagement with male household members and community leaders. Paired implementation enhanced service utilization and helped shift household norms. However, challenges persisted, including gender selection barriers, literacy gaps, and limited institutional support for paired training and supervision.

**Conclusion:**

The male–female VHV/VHWs model may enhance access to MCH services by engaging men and women in complementary roles tailored to local contexts. To maximize its potential, institutional commitment is needed to formalize pair-based training, address gender barriers in recruitment, and strengthen community- and policy-level support systems for CHWs. Given the 2023 PHC policy, embedding gender-sensitive and context-specific approaches into guidelines and training manuals may be critical for aligning service delivery with sociocultural realities and ensuring responsiveness to the unique needs of remote communities.

## Background

Community health workers (CHWs) are essential to improving access to primary health care (PHC) and achieving universal health coverage. According to the World Health Organization, CHWs are lay health personnel selected from the communities in which they work, accountable to those communities, and supported by the broader health system [1]. Although CHWs often have lower levels of formal education and clinical training compared to professional health care providers, they form a crucial link in the health workforce. Evidence from both low- and middle-income as well as high-income countries demonstrates their effectiveness in expanding access to essential health services, particularly among underserved and marginalized populations [2–4]. Their responsibilities span a wide range of activities, including health education, screening, facilitating access to PHC services, and engaging in disease-specific interventions such as medication treatment and basic first aid [5]. In carrying out these tasks, CHWs’ intimate knowledge of local cultural norms and social structures, along with their closeness to the community, constitutes a vital asset that enables them to provide contextually appropriate care [5]. This unique positioning enables CHWs to address sociocultural barriers to care, which is particularly important for reducing health disparities, including for maternal and child health (MCH), and for strengthening equity-oriented health systems in marginalized settings [4, 6].

Numerous studies have shown that CHWs have the potential to contribute to reducing maternal and neonatal morbidity by promoting timely access to essential health services at the community level. A systematic review indicated that newborn home visits by CHWs were associated with a 38% decrease in neonatal deaths in South Asia [7]. Additionally, several studies have shown that CHWs contribute to improving women’s health during the perinatal period through the early detection of pregnancy-related complications, such as pre-eclampsia and postpartum hemorrhage, and the referral of affected individuals to appropriate health facilities [8, 9]. The World Health Organization issued guidelines in 2015 advocating task-shifting strategies to CHWs, with the aim of enhancing their involvement in delivering essential care at the community level [1].

Despite the contributions of CHWs to MCH, their effectiveness in MCH is influenced by structural and contextual factors, including gender-related barriers. Gender dynamics affect how CHWs are perceived, how they perform, and how they engage with communities. From the CHWs’ standpoint, several gender-related factors can influence how effectively they function in the field. In some settings, female CHWs may face mobility constraints due to concerns about personal safety, religious or cultural norms that prohibit women from walking alone, or the need to obtain permission from family members to travel [10–12]. From the community’s perspective, gender also influences the quality of interaction and trust building. Particularly in the domain of reproductive, maternal, and child health, female CHWs often have greater access to women, enabling more open communication about sensitive topics such as family planning, breastfeeding, and postpartum care [13, 14]. For example, a study conducted in Tanzania suggested that male CHWs may feel more comfortable discussing sexual and reproductive health issues with men; however, due to prevailing gender dynamics and social norms, they are often less accepted by women when addressing these topics, which limits their impact on the use of reproductive and maternal health services [13]. Conversely, while female CHWs may be more likely to receive early pregnancy disclosures from women, they frequently encounter difficulties in engaging with male partners and senior family members—challenges that are similarly shaped by gendered power relations and sociocultural expectations within the household and community [11]. In such contexts, working in male–female pairs may help ensure safety, legitimacy, or community acceptance [11–13, 15].

Similar to many low- and middle-income countries, the Lao People’s Democratic Republic (Lao PDR) continues to experience challenges in providing PHC, particularly in remote and ethnically diverse areas [16, 17]. Structural barriers such as limited human resources, constrained financing, poor infrastructure, and geographic isolation contribute to ongoing health disparities among ethnic minority populations living in mountainous and border regions [18, 19]. The 2023 Lao Social Indicator Survey (LSIS III) highlights persistent urban–rural inequities in MCH. For instance, the infant mortality rate in rural areas is estimated to be 2.6 times higher than in urban settings [20]. Additionally, while 86.7% of urban women received four or more antenatal care (ANC) visits, only 66.3% of rural women received four or more ANC visits. Institutional delivery rates were lower in rural areas (75.8%) than in urban ones (83.2%) [20]. Similar gaps were observed in family planning and breastfeeding, with rural women consistently reporting lower coverage and poorer practices compared to urban women [20]. Although health services are available, they remain underutilized due to poverty, language barriers, and entrenched gender norms that restrict women’s autonomy in making health-related decisions [21]. In response to these challenges, CHWs have been identified as key facilitators in improving access to maternal health services. A study conducted in remote and ethnic communities in Lao PDR reported a positive association between higher levels of trust in CHWs and increased utilization of postnatal care among mothers [22]. The study suggests that, alongside enhancing maternal trust in VHVs/VHWs, it may be important to systematically address restrictive gender norms through targeted male engagement strategies.

In the Lao PDR, CHWs, known as village health workers or village health volunteers (VHWs/VHVs), serve as the cornerstone of community-based PHC, particularly in remote and hard-to-reach areas. National policy ensures that each village should have at least one VHW/VHV and is responsible for delivering primary health services including MCH [23]. In Xepon District, an ethnically diverse, mountainous area in Savannakhet Province, approximately 90% of VHWs/VHVs are male [24]. This gender imbalance is largely attributed to national selection criteria that require basic literacy and completion of primary education. These qualifications have historically been inaccessible to many women in remote ethnic minority communities because of limited access to formal schooling. While male VHWs/VHVs perform essential functions in health promotion, sociocultural norms in Xepon District limit the extent to which they can engage effectively with women on MCH issues such as ANC, birth preparedness, family planning, and breastfeeding. To address this limitation, a pilot activity led by the provincial health department was launched in 19 villages in the district in 2017. In each village, one male VHV and one newly selected female VHV were paired, resulting in a total of 38 volunteers. The intervention was implemented until 2019, suspended for approximately three years during the COVID-19 pandemic, and subsequently reinitiated in 2023. Currently, the model remains a provincially led initiative and has not yet been scaled up in other areas. Of the 14 health centers operating under the Xepon District Hospital, four were purposively selected based on their accessibility. From the catchment area of each selected health center, two to seven villages were identified in consultation with local health authorities, considering logistical feasibility and population needs. The pilot project introduced a male–female paired VHW/VHV model to strengthen MCH outreach. Under this model, female VHV/VHWs were selected in each village and trained to collaborate with existing male VHWs/VHVs. Together, they conducted home visits for pregnant and postpartum women, providing maternal health education and mobilizing community members during outreach visits by health center staff. Due to widespread literacy limitations among the female VHVs/VHWs, the male VHWs/VHVs had responsibility for completing monitoring forms and recording health information during home visits. A study conducted in these pilot villages found that mothers who received home visits from paired VHVs reported lower Edinburgh Postnatal Depression Scale (EPDS) scores compared with those visited by single male VHVs, suggesting potential benefits of the paired approach for maternal mental health [25]. Nevertheless, how CHW engagement affects maternal health outcomes remains poorly understood and warrants further investigation.

MCH indicators in Xepon District remain suboptimal despite national progress, with gaps in antenatal care, family planning, and breastfeeding particularly evident in remote, ethnically diverse settings. As approximately 90% of VHVs are male, gender norms may constrain their ability to engage women on sensitive MCH issues. To address this challenge, a provincial pilot introduced male–female VHWs/VHVs pairs to improve outreach and promote gender-sensitive service delivery. However, little is known about how these pairs function in their communities or how they are perceived by households and community members. This study, therefore, aimed to explore the functioning of male–female VHWs/VHVs pairs in Xepon District, focusing on their roles, interactions, and community responses.

The findings may inform future policy and programmatic decisions to strengthen gender-sensitive health interventions in underserved, culturally diverse contexts.

## Methods

### Study site

The survey was conducted in 19 model villages in Xepon District, one of the poorest and most remote areas in Lao PDR. Xepon District, in the eastern part of Savannakhet Province near the border with Vietnam, is a rural and mountainous area located approximately 600 km from the capital city, Vientiane. The district comprises 88 villages and has a population of about 66,995 people, of whom around 75% belong to ethnic minority groups, mainly Phou Thai, Tri, and Mangkong (Xepon District Governor’s Office, unpublished data, 2024). In addition to the Lao national language, Phou Thai and Brou languages (including Tri and Mangkong dialects) are widely spoken. While individuals who have attended school generally understand and speak Lao, ethnic minority women with limited schooling often have low literacy and may not speak Lao fluently. Because the provincial pilot intervention purposively included Tri villages, participants from these communities were not excluded from the study, and in cases where Brou-speaking participants were involved, a Brou-speaking research assistant provided complementary interpretation support during interviews. The district’s health service infrastructure includes 14 health centers and one district hospital [24]. Each village is administratively assigned to the nearest health center to receive PHC services.

### Study design and participants

This study used a qualitative descriptive design to explore the implementation and perceived effects of the male–female paired VHW/VHV model. A purposive sampling was used to obtain a diverse range of views. Participants included women who had given birth within the previous 12 months and received support from VHWs/VHVs pairs during pregnancy, as well as their husbands or partners. Individuals who had experienced fetal loss, stillbirth, neonatal death, or infant death during the relevant pregnancy period were excluded to minimize potential emotional distress due to the absence of on-site bereavement support and reliable referral pathways at the time of fieldwork. In addition, currently active VHWs/VHVs engaged in paired arrangements, health center staff supervising VHWs/VHVs activities, and personnel from district and provincial health offices involved in MCH programming were also included to provide system- and policy-level insights. In total, 42 participants were purposively sampled to capture diverse perspectives related to maternal and child health in Xepon District. The sample included women (*n* = 16), men (*n* = 4), VHWs/VHVs (*n* = 10), village leaders (*n* = 2), health center staff (*n* = 4), district health officials (*n* = 3), and provincial health officials (*n* = 3).

### Data collection

Data collection was conducted from June to October 2024 using semi-structured interviews, guided by topic outlines specifically designed to capture each participant group’s views on the paired VHW/VHV model. Participants were selected through purposive and snowball sampling methods to ensure the inclusion of individuals with relevant insight and experience. The research team developed separate interview guides for mothers, their spouses, VHWs/VHVs, health center staff, district health officials, and village leaders. All interviews addressed common themes including perceptions of the male–female VHW/VHV pairing model, its acceptability within the community, and perceived benefits and challenges related to MCH service delivery. In addition to these shared areas of inquiry, participant-specific topics were explored based on the interviewee’s role. These included, for example, experiences with service utilization and support during the perinatal period, observed changes in household and community health behaviors, gender-related dynamics in collaboration, and perceptions of supervision and coordination. The guides were tailored to capture both individual and system-level insights relevant to the implementation and effects of the paired VHW/VHV model. The interview guides were initially developed in English, then translated into Lao and checked with district hospital and health center staff to ensure linguistic and contextual appropriateness. Prior to initiating formal data collection, revisions to both content and translation were made based on feedback. Two trained research assistants supported the data collection process. The study facilitator explained the research objectives and procedures to each participant, and written informed consent was obtained before all interviews. Although the interviews were conducted in Lao, some mothers had limited proficiency in the Lao language. In such instances, a Brou-speaking research assistant provided interpretation to facilitate accurate communication and participant comprehension. Transcripts were primarily translated from Lao into English for coding. For Brou-speaking participants, interviews were first translated into Lao and then into English. For the other interviews, the initial transcription and translation were performed by an experienced external translator proficient in both Lao and English. To ensure accuracy and contextual fidelity, the bilingual research member reviewed the translated transcripts against the original recordings. Each interview lasted approximately 15 to 40 min.

### Data analysis

The translated transcripts were uploaded into MAXQDA 24 (https://www.maxqda.com/new-maxqda-24) for data management and analysis. For mothers, saturation was considered reached when repeated interviews no longer generated new codes or concepts [26, 27]. For other participant groups (husbands, village leaders, VHV/VHWs, and health staff), the number of eligible individuals was limited, and all available participants were interviewed. The data were analyzed using inductive thematic analysis, following the six-phase approach proposed by Braun and Clarke [28]. We adopted an inductive, data-driven thematic analysis given the exploratory aim and limited prior literature in this setting. No priori theory or framework was imposed, consistent with Braun and Clarke’s view that inductive thematic analysis derives themes directly from the data rather than from pre-existing theoretical frames [28]. We first developed an overview of the open codes, focusing on community changes after the introduction of female VHV/VHWs and the formation of male–female pairs. The interview transcripts were reviewed multiple times, resulting in the identification of 122 initial codes. These codes were then consolidated into 38 broader categories through an iterative coding and refinement process. To strengthen the validity of the analytical process, member checking was conducted within the research team through collaborative discussions of preliminary codes and emerging themes to promote consistency and reduce interpretive bias. Additionally, a third researcher, not involved in the initial coding, reviewed all themes and illustrative excerpts for coherence, further enhancing the credibility of the findings. Any discrepancies in interpretation were resolved through collective deliberation until an agreement was reached on the final thematic framework.

## Results

The analysis produced 122 initial codes, which were consolidated into 38 categories. These categories reflected common patterns in the participants’ views and were then grouped into four main themes. The themes were: (1) “Building Trust and Comfort Through Gender-Matched Interactions”; (2) “Gender-Specific Responsibilities and Task-Sharing between Paired VHVs”; (3) “Strengthening Family Support and Health System Linkages through Female VHV/VHWs Engagement”; and (4) “Challenges and Support Needs for Strengthening the VHV Program.”

### Theme 1: building trust and comfort through gender-matched interactions

This theme illustrated how female VHV/VHWs enhanced culturally appropriate and gender-sensitive health engagement in remote, ethnic minority communities. Female VHV/VHWs were widely perceived as approachable, empathetic, and trustworthy by the pregnant and postpartum women. Participants reported that their presence helped overcome sociocultural barriers that often prevent open discussion of reproductive health issues with male VHVs.

“Female VHV asked me how I was doing whenever she saw me during my pregnancy. She makes me relaxed when we talk. She is like a friend… if women talk to male VHV, many of us will be shy and unable to get a consultation.” (Mother)

In settings where gender norms discourage direct communication between men and women, female VHV/VHWs created a comfortable environment in which women could share reproductive health concerns freely. Their access to women’s daily spaces aligned with community expectations and enabled ongoing follow-up.

“When she sees pregnant women, even just around the village, she always reminds us to be careful and not to do hard work or carry heavy things. She cares for everyone in the village.” (Mother)

“If a male VHV wants to find out who is pregnant, he can’t ask directly… But a female VHV can go and ask them directly, which makes things much easier.” (Health center staff)

Through their trusted social position and gender-congruent communication, female VHVs/VHWs facilitated initial contact with pregnant women, enabling early access to information on ANC and encouraging subsequent engagement with the formal health system.

### Theme 2: gender-specific responsibilities and task sharing between paired VHVs

This theme examined the gender-specific division of responsibilities between the male–female VHVs/VHWs. Female VHVs/VHWs were primarily responsible for identifying pregnancies early, often through informal interactions within women’s social spaces and providing maternal health information and emotional support.

“I do monthly follow-ups and ask women directly. Sometimes I ask their friends, and they might say, ‘Oh, she might be pregnant—maybe two or three months already.’ Then I go visit her, and she tells me. Usually, they tell me around two or three months.” (Female VHV/VHW)

“Back then, no one encouraged or convinced pregnant women to go to the hospital or health center for ANC, so the number of ANC visits was quite low. But now, we have paired VHVs in each village. The number of ANC visits is increasing. In addition, before, most women came late—usually after 20 weeks. But now, women are starting to come earlier, like around 10 weeks.” (District health office staff)

However, in communities where male household members hold primary decision-making power, female VHVs/VHWs often faced challenges in advocating for MHC services. They reported hesitancy or limited capacity to influence male heads of households, particularly regarding service uptake, participation in health education, or child vaccination.

In such situations, male VHVs were more likely to be accepted as intermediaries. Their perceived authority and social position allowed them to communicate effectively with husbands, village leaders, and health center staff. Their support often proved essential in legitimizing the health-related decisions initiated or facilitated by the female VHVs/VHWs.

“In our culture, it's not acceptable for a man to sit close and talk with a woman who isn't his wife, and the same goes for a woman with a man who isn't her husband. Sometimes, a husband doesn’t want his wife to participate in health education or receive home visits from VHVs. In those cases, the male VHV has more influence and can explain why it’s important for her to take part in health activities. In our culture, people tend to listen to the husband. If he doesn’t allow her to go, she won’t go. That’s why male VHVs often need to step in and speak with the husbands.” (Health center staff)

Together, the paired VHV model enabled culturally appropriate and gender-sensitive delivery of health services by leveraging the comparative strengths of both the male and female volunteers.

### Theme 3: strengthening family support and health system linkages through female VHV/VHWs engagement

This theme highlighted how the inclusion of female VHVs/VHWs contributed to increased household support for MHC, while also strengthening coordination with the health system.

In settings where male involvement in reproductive health was traditionally minimal, the visible and consistent presence of female VHVs/VHWs helped shift household dynamics. Husbands who observed VHVs supporting their wives became more open to guidance, and household-level discussions on ANC, birth preparedness, and family planning became more common.

“Before, husbands didn’t know how to help. But now, when both VHVs visit, men also listen and start to understand more about pregnancy.” (Mother)

“My husband started helping more when he heard the female VHV explaining why I needed to rest during pregnancy.” (Mother)

“Some families didn’t let mothers breastfeed because they had to go back to work right after delivery, so the children weren’t fed properly. But things have changed since the male–female VHVs/VHWs pairs started working. Now, fathers are more involved in childcare and better understand the importance of health care and vaccination.” (Health center staff)

Beyond the household level, female VHVs/VHWs acted as important intermediaries between communities and the formal health system. Through regular interactions with families, they were able to identify pregnancies early, reinforce health messages, and facilitate timely referrals. Health center staff noted improved communication and follow-up, which enhanced continuity of care.

“Our work has become easier since female VHVs started working. Back then, if there was a problem with reporting, I had to go to the village by myself to get the information. But now, I just call the VHVs, and they can give me the details. The female VHVs have up-to-date information about the women and children in the village.” (Health center staff)

“Before the outreach, we give a list of target families to the paired VHVs, and they divide the work. The female VHV lets the mothers know to come to the meeting point, while the male VHV takes care of other tasks. In the past, community members weren’t very cooperative when we came, but now everything is more organized.” (Health center staff)

Health providers also reported increased utilization of services in areas covered by paired VHVs, which included ANC attendance, institutional deliveries, child immunizations, and more frequent involvement of husbands during ANC visits and childbirth, reflecting more inclusive MCH practices.

### Theme 4: challenges and support needs for strengthening the VHV program

This theme highlights that despite the demonstrated benefits of the paired approach, both male and female VHVs faced systemic barriers. While the community generally received male–female VHW/VHV teams positively and their complementary roles improved service delivery, persistent gender norms continued to shape and sometimes restrict their effectiveness. For instance, female VHV/VHWs may face limitations due to household responsibilities or childcare demands, making it more difficult for them to participate in meetings or training sessions. Additionally, lower literacy levels among women can result in male VHVs being more frequently selected for externally funded projects.

“We expect that pairing male and female VHVs will improve their effectiveness. But in reality, female VHVs often have less knowledge and face more challenges. For example, it's harder for them to travel, attend trainings, or submit reports. Male VHVs usually have fewer restrictions and can handle these tasks more easily. If female VHVs had more knowledge and training, the work could be more balanced and effective.” (District health office)

Supportive supervision was provided by health staff, but it was not always systematic. District health staff reported that collaboration with community organizations such as the women’s and youth unions and village authorities was occurring in several villages. They described these collaborations as improving community involvement in VHV activities and helping VHVs address challenges in their work.

“Following the training, VHVs must also engage with village leaders and community members. During village meetings, VHVs are encouraged to provide guidance and mobilize support. It’s not just the VHVs working alone; the community needs to collaborate to ensure the success of these efforts.” (District health office)

“When VHVs and health center staff have a problem with villagers, we try to support them. If we can't solve it ourselves, we involve others—like the women's union, youth union, or sometimes even the education sector.” (District health office)

Concerns remained regarding the sustainability and inclusivity of the VHV program in the selection of female VHV/VHWs. In many areas, village leaders made the final decision, and district health offices often deferred to them. Health staff noted that this practice limited women’s opportunities to volunteer, particularly in communities where gender norms discouraged self-nomination.

“It’s good to have male VHVs, but we want more female VHVs to be actively involved. Still, there are things we can’t intervene in directly like the selection process. That’s up to the community leader. We only check if the person meets the requirements, not whether it should be a man or a woman.” (District health office)

“We still need to make efforts to recruit and select the right individuals. It’s achievable, but it requires a collaborative effort between the government and the communities to ensure that the selection process is thorough and that the chosen individuals are capable and committed.” (Provincial health office)

Overall, the findings showed that sustaining the paired VHV program was challenged by limited institutional support, restricted training opportunities for female VHVs, and community-led selection processes that often lacked transparency and inclusivity.

## Discussion

This study qualitatively explored the male–female VHVs/VHW model in remote ethnic minority communities of Lao PDR. The findings show that this paired approach improved maternal health support and fostered positive community perceptions. Female VHV/VHWs played a critical role in reaching women in culturally acceptable ways, providing emotional support, health education, and timely referrals throughout pregnancy and after childbirth. Their trusted presence facilitated early identification of pregnancies and follow-up care. Male VHVs, often perceived as community leaders, engaged men and village leaders, supported logistics, and helped legitimize maternal health messages. Together, these complementary roles helped overcome sociocultural barriers, increase household involvement, and improve ANC service utilization (Table 1).

**Table 1:** Demographic of the participants

Group	Number of participants	Age range (years)	Educational attainment (*n*)	Years of experience
Women	16	17–35	No education (10)	N/A
			Elementary (3)	
			Middle school (1)	
			High school (1)	
			Higher (1)	
Men	4	DK	DK	N/A
Village leaders	2	49–62	Elementary (1)	30–32
			Middle school (1)	
Female VHV/VHWs	7	19–70	Middle school (6)	1–28
			High school (1)	
Male VHV	3	46–60	Middle school (3)	6–14
Health center staff	4	36–50	Higher (4)	6–14
District health office	3	46–51	Higher (3)	22–26
Provincial health office	3	51–54	Higher (3)	22–35

A major strength of this study is that it is among the first to examine the paired VHV model, providing context-specific insights from multiple stakeholders. The use of a Brou-speaking research assistant minimized language barriers and strengthened credibility. However, the findings are context-specific and may not be generalizable to other settings. Additionally, participants who experienced fetal or neonatal loss were excluded, potentially limiting perspectives on negative care experiences.

Our findings are consistent with research from South and Southeast Asia showing that gender-matched CHW strategies improve service acceptability [29, 30]. This study extends the literature by describing the collaborative implementation of paired VHVs in the sociocultural setting of Lao PDR and by documenting context-specific barriers—such as reliance on village leaders for selection and limited literacy among female VHVs—that are less frequently reported in other settings.

Integrating gender-responsive and community-engaged approaches into CHW programs provides a strategic pathway for strengthening PHC and advancing progress toward universal health coverage. Experience from India’s ASHA (accredited social health activist) program illustrates that gender-aligned service delivery can foster trust and improve access to maternal and reproductive health services, especially among women in culturally conservative settings [29]. Previous studies indicate that CHW programs attentive to gender dynamics and active community engagement can enhance equity, strengthen health systems, and improve coverage of care [29, 31]. Although the paired approach was effective in the cohesive and culturally homogeneous settings examined here, the findings caution against assuming its universal applicability. In more urbanized communities, where social cohesion and gender norms differ significantly, alternative approaches may be required. Glenton et al. highlight that factors such as cultural context, gender norms, education levels, and system support influence the effectiveness and sustainability of CHW programs, and emphasize the need for contextually grounded planning [32]. Building on this, Kok et al. stress the importance of aligning intervention design with broader structural and systemic conditions—such as health system organization, resource availability, and policy environments—to enhance CHW performance [33]. Together, these insights underscore that while gender-matched CHW strategies can be effective, their design and scale-up should be grounded in contextual evidence from comparable settings to ensure relevance and impact across diverse environments.

Despite the model’s strengths, the study also identified the following implementation challenges. One major issue was the selection process for female VHV/VHWs. The selection of VHVs is primarily determined by individual village leaders, with limited coordination or oversight from the formal health system. This practice may prioritize candidates with greater social visibility or personal connections, which can result in less attention being given to their actual competence or genuine motivation. Such practices risk excluding women with the potential to succeed in the role, particularly those who may be less publicly engaged due to household responsibilities or prevailing gender expectations. Transparent and participatory recruitment processes involving both community leaders and residents have been shown to positively influence CHW motivation and performance when CHWs are selected based on social proximity, trustworthiness, and personal qualities valued by the community [32]. Several studies emphasize that a community-rooted selection approach, where lay health workers are embedded within the social fabric of their communities through shared norms or valued traits such as empathy and respect, can help foster strong, trusting relationships with recipients [33, 34]. In the Lao context, institutionalizing the selection process with clear guidelines and shared responsibility between village leaders and health center staff may help ensure fairness, improve volunteer performance, and increase program credibility. Encouraging the active involvement of district- and provincial-level staff in overseeing recruitment and promoting women’s participation through public endorsement can further reinforce gender equity in community health governance.

Another challenge is that the limited formal education of many female VHVs/VHWs underscores the need for sustained informal training to support their effectiveness. However, one-off training workshops often fail to meet the needs of volunteers when they are not tailored to local realities. As Ghanekar argues, *“one-size-fits-all”* capacity-building models are inadequate, especially across diverse rural and urban settings, where contextual differences shape the competencies required of CHWs [35]*.* This highlights the importance of adaptive and context-specific training approaches that can respond to the unique challenges faced by VHVs/VHWs in remote, low-resource communities. In such cases, informal learning such as peer mentoring, experiential learning, and on-the-job coaching can provide flexible, context-specific support aligned with practical tasks [36–38]. For instance, Willock et al. demonstrated that peer-led training significantly enhanced CHWs’ knowledge, confidence, and capacity to deliver targeted health education in underserved communities [37], while the Care Groups model showed that structured peer support and regular group learning can sustain motivation and improve maternal and child health outcomes in resource-constrained settings [38]. Strengthening informal learning opportunities may enhance both the technical performance of female VHVs/VHWs and their involvement in leadership and decision-making processes, contributing to more inclusive and gender-equitable community health systems.

Although recent Lao health policies emphasize the promotion of female volunteers, they do not explicitly recognize the male–female paired VHW/VHV model [23]. As a result, training programs tend to emphasize individual roles, with less attention given to developing the collaborative skills necessary for effective task sharing and joint engagement with families. This policy gap may limit the full potential of the paired approach, despite its demonstrated benefits in enhancing cultural acceptability and service reach. Glenton et al. emphasize that gender norms, institutional support, and policy alignment are critical for sustaining CHW programs [32]. Incorporating the gender-sensitive paired model into formal policy and training frameworks would help institutionalize its practice, ensuring its scalability and long-term impact.

## Limitations

This study has several limitations. First, the findings are context specific, reflecting experiences from Brou-speaking rural communities in Lao PDR, and may not be generalizable to other settings. Nonetheless, they may offer insights for similar ethnolinguistic and remote populations. Language posed a potential challenge, as interviews were conducted in Brou languages, translated into Lao by the research assistant, and then into English for analysis. Although the use of a Brou-speaking research assistant helped minimize misinterpretation, multiple layers of translation may have led to a loss of nuance in participants’ narratives. Participants who experienced adverse outcomes such as stillbirth or neonatal death were excluded from the study, and their perspectives might have provided valuable insights into the limitations of VHVs/VHWs support in situations of poor maternal and child health outcomes. Additionally, social desirability bias may have influenced responses on sensitive topics such as gender norms and household dynamics, despite efforts to build rapport and ensure confidentiality. Even with these limitations, the study offers valuable insights into gendered VHV roles and the sociocultural factors shaping MCH service delivery in underserved contexts.

## Conclusion

The present qualitative study indicates that the functioning and perceived contributions of the paired VHV/VHW model may be shaped by system-level conditions that extend beyond individual volunteers. Embedding gender-transformative approaches within supportive structures such as transparent recruitment, culturally responsive training, and mentorship-based supervision may help strengthen implementation. As the Lao VHV program expands, stronger institutional commitment could support longer-term sustainability and more equitable reach. Given that a national PHC policy was established in 2023, embedding gender-sensitive and context-specific approaches into legal guidelines and training manuals may be critical for aligning service delivery with local sociocultural realities. Such integration could further facilitate the scale-up of the paired service model and ensure its responsiveness to the unique needs of remote communities. Further research is necessary to assess transferability and effects across diverse settings.

## Data Availability

No datasets were generated or analyzed during the current study.

## Abbreviations

ANC: Antenatal care
CHW: Community health worker
DK: Don’t know
UHC: Universal health coverage
MCH: Maternal and Child Health
PHC: Primary health care
VHW/VHV: Village health worker/village health volunteer
